# A Japanese patient with neonatal biotin-responsive basal ganglia disease

**DOI:** 10.1038/s41439-022-00210-z

**Published:** 2022-09-29

**Authors:** Mizuki Kobayashi, Yuichi Suzuki, Maki Nodera, Ayako Matsunaga, Masakazu Kohda, Yasushi Okazaki, Kei Murayama, Takanori Yamagata, Hitoshi Osaka

**Affiliations:** 1 Division of Pediatrics, Jichi Medical University, Tochigi, Japan; 2 Department of Pediatrics, Fukushima Medical University School of Medicine, Fukushima, Japan; 3 Center for Medical Genetics, Department of Metabolism, Chiba Children’s Hospital, Chiba, Japan; 4 Diagnostics and Therapeutics of Interactable Diseases, Interactable Disease Research Center, Graduate School of Medicine, Juntendo University, Tokyo, Japan

**Keywords:** Genetics, Neuroscience

## Abstract

Biotin-responsive basal ganglia disease (BBGD) with *SLC19A3* mutation was first reported in 1998, and over 30 mutations have been reported. We report a neonatal BBGD case with sudden-onset feeding difficulty and impaired consciousness. Encephalopathy resolved after the initiation of biotin and thiamine treatment. Genetic testing revealed a novel heterozygous mutation [c.384_387del, p.Tyr128fs];[c.265 A > C, p.Ser89Arg] in *SLC19A3*. Early treatment for BBGD is essential, especially with onset in the neonatal or early infancy period.

## Introduction

Biotin-responsive basal ganglia disease (BBGD) is an autosomal recessive disorder that causes catastrophic subacute metabolic encephalopathy^1–3^. BBGD includes a wide variety of neurological phenotypes, including early-infantile Leigh-like encephalopathy, classic recurrent subacute encephalopathy, and adult-onset Wernicke-like encephalopathy^1^. Patients with the most severe phenotype present with poor feeding, vomiting, and acute encephalopathy with severe lactic acidosis. BBGD is now recognized as thiamine metabolism dysfunction syndrome 2 and is caused by variants of thiamine transporter type 2 (ThTR-2), which is encoded by *SLC19A3* (#606152)^1^.

More than 30 mutations in *SLC19A3* have been reported^3^. Early-infantile severe encephalopathies in BBGD are caused by nonsense or frameshift variants. Therefore, loss of ThTR2 function is thought to cause this disease^2,3^. In most cases, early-infantile encephalopathy is associated with poor outcomes or death, even after supplementation with biotin and thiamine^1^. We report a neonatal case of BBGD in which treatment with biotin and thiamine improved clinical symptoms and reduced further deterioration. Overall, early diagnosis and initiation of biotin and thiamine treatment is crucial for this treatable condition.

**Fig. 1:**
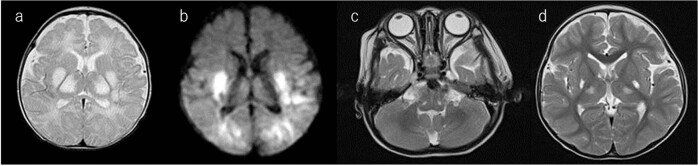
The patient’s magnetic resonance images. **a**, **b** Acute-phase brain MRI showed high-intensity signals in the bilateral thalamus, putamen and globus pallidus. **c**, **d** Follow-up brain MRI showed high-intensity signals in the bilateral thalamus, liquefaction of the dorsal putamen, and atrophy in the cortex, subcortical white matter, and white matter as sequelae of neonatal encephalopathy.

The patient was a 6-year-old boy born via normal delivery (body weight, 2746 g; height, 54.0 cm) at 39 weeks of gestation to nonconsanguineous parents without any specific family history. His mother had no remarkable pre- or perinatal history. His parents brought him to a secondary care hospital on the 28th day after birth due to sudden-onset feeding difficulty and impaired consciousness. Brain CT and magnetic resonance imaging (MRI) revealed high-intensity signals in the bilateral thalamus, putamen and globus pallidus (Fig. 1). Metabolic encephalopathy was suspected, and he was transferred to a tertiary care center on the same day for more intensive treatment and diagnostic tests. His CSF levels of lactate and pyruvate were slightly elevated (lactate/pyruvate: CSF 2.6/0.15 mmol/L, normal range of lactate <1.8 mmol/L, CSF L/P ratio 16.7, normal range <15). Neonatal tandem mass screening showed no abnormalities. Urine organic acid analysis revealed elevated dicarboxylic acid (adipic acid, suberic acid, sebacic acid), 3-OH-butyrate and acetoacetate levels. After 3 weeks of hospitalization, the patient was diagnosed with Leigh syndrome based on the MRI findings and elevated lactate level. Vitamin treatment (thiamine 6.6 mg/kg, ascorbic acid 78.7 mg/kg, tocopherol 7.9 mg/kg, and carnitine 65.6 mg/kg) was initiated. He had an episode of acute bronchitis at 4 months and febrile status epilepticus at 10 months, but there was no evidence of elevated lactic acid and pyruvate or transaminases at either of these times. His electroencephalogram was normal, and phenobarbital suppository was initiated when he developed fever. As the clinical course and MRI images were suggestive of BBGD, biotin 10–15 mg/kg/day was initiated at this time in addition to an increased dosage of thiamine 10–20 mg/kg/day, and no recurrence of encephalopathy or other crisis occurred. After initiating biotin and thiamine, he gradually gained eye tracking and smiling and developed shyness. After obtaining informed consent, target sequencing was performed for the patient using a modified version of a previously described method^4^. Briefly, indexed genomic DNA (gDNA) libraries were prepared from gDNA of the patient’s blood, and target regions, including 264 genes and the entire mitochondrial genome, were captured using SureSelect Custom Target DNA Enrichment kits (Agilent Technologies) in accordance with the manufacturer’s protocol; 150-bp paired-end read sequencing was performed using MiSeq (Illumina). We used Sanger sequencing to confirm the mutations identified and assess the carrier status of unaffected family members. PCR direct sequencing was performed using a BigDye v3.1 cycle sequencing kit (Thermo Fisher Scientific) and Genetic Analyzer. A compound heterozygous *SLC19A3* mutation, NM_025243.4: [c.384_387del,p.Tyr128fs];[c.265A>C,p.Ser89Arg], was identified in the patient. A novel truncating mutation [c.384_387del,p.Tyr128fs] inherited from the mother appears to be pathogenic. A variant [c.265A>C,p.Ser89Arg] inherited from the father is likely pathogenic because it has already been reported in four cases^3,5,6^; it is predicted to be deleterious/damaging by 4 of 5 variant prediction programs (Supplementary Table 1). We classified the variants according to the ACMG_AMP classification guideline^7^. [c.384_387del,p.Tyr128fs] is thought to be pathogenic in PVS1 (the variant is thought to cause early truncation resulting in loss of function), PM2 (extremely low frequency), and PP4 (not been reported, but the phenotype of the compound mutations with other clinical BBGD cases is very specific). [c.265A>C,p. Ser89Arg] is likely pathogenic in PM2 (extremely low frequency; allele frequency = 0.00024 in the Japanese population; GnomAD exomes homozygous allele account = 0), PM3 (the other allele is thought to cause early truncation resulting in loss of function, and the phenotype is thought to be a recessive disorder), PP3 (multiple lines of computational evidence of a deleterious effect of the variant are provided in Supplementary Table 1), and PP4 (reported in four cases with LS or BBGD^3,5,6^, clinically specific phenotypes for the gene). Sanger sequencing for the parents and patient confirmed the mutations identified (Supplementary Fig. 1). We did not find any SNPs that account for the developmental delay, only the *SLC19A3* aberration. The patient’s follow-up brain MRI showed high-intensity signals in the bilateral thalamus, liquefaction of the dorsal putamen, and atrophy in the cortex, subcortical white matter, and white matter as sequelae of neonatal encephalopathy (Fig. 1). The patient now smiles and follows objects with his eyes. However, he displays severe intellectual disability without the ability to form words. His muscle tone is hypotonic, and tendon reflexes are elevated with a positive Babinski sign. The patient cannot hold his head or roll over. Due to swallowing difficulty, he is fed by gastrostomy.

Thiamine, known as vitamin B1 or aneurin, is an essential water-soluble vitamin that is not produced by humans^8^. It must be obtained orally and is absorbed through the intestinal brush border. Thiamine transporter type 2 encoded by *SLC19A3* is expressed in the small intestine in response to uptake of thiamine by cells^9^. Thiamine malabsorption due to loss of thiamine transporter type 2 causes BBGD. Thiamine pyrophosphate (TPP), which accounts for 80% of thiamine in the entire body, is produced from thiamine and binds to the pyruvate dehydrogenase complex as a coenzyme^10^. If TPP is not synthesized or if it is undersynthesized, acetyl-CoA from pyruvate in the TCA cycle is not produced, leading to energy depletion in cells^10^.

Biotin is a coenzyme for carboxylases, including pyruvate carboxylase in the TCA cycle, and helps to produce oxaloacetic acid to bypass the generation of acetyl-CoA from pyruvate^11^. This pathway is thought to act as an alternative for the generation of acetyl-CoA under thiamine-deficient conditions.

To our knowledge, 10 cases of neonatal-onset BBGD have been reported to date (Table 1). Eight cases involved early truncation mutations. The novel mutation [c.384_387del,p.Tyr128fs] in our patient is also thought to cause early truncation of the protein, leading to loss of function. The recurrent [c.265A>C,p.Ser89Arg] mutation, in another allele has been reported in patients with severe Leigh syndrome, with onset within 1 year of birth^12^ (Supplementary Table 1).

**Table 1:** Related demography and outcome by mutations of neonatal encephalopathy patients.

Reference	Mutations	Age at onset	Consanguinity	treatment	Outcome
This case	c. 384_387del, p.(Tyr128fs)	28 days	No	Biotin and thiamine	Bedridden
	/c.265A>C, p.(Ser89Arg)				
Yamada et al.^14^	c.958G>C, p.(Glu320Gln)	1M	Yes	NP	Bedridden
	/c.958G>C, p.(Glu320Gln)				
Perez-Duenas et al.^15^	c.68G>T, p.(Gly23Val)	1M	ND	Biotin and thiamine	Gait
	/c.68G>T, p.(Gly23Val)				
Gerards et al.^16^	c.20C>A, p.Ser7Ter	1M	No	NP	Death
	/c.20C>A, p.Ser7Ter				
Haack et al.^17^	c.982del, p.(Ala328Leufs*)	neonatal	Yes	NP	Death
	/c.982del, p.(Ala328Leufs*)				
Haack et al.^17^	c.982del, p.(Ala328Leufs*)	18 days	Yes	Biotin and thiamine	Normal
	/c.982del, p.(Ala328Leufs*)				
Kamasak et al.^18^	c.623_624insA, p.(Ser179fs)	30 days	No	Thiamine	Responds to mother
	/c.623_624insA, p.(Ser179fs)				
Kamasak et al.^18^	c.620delinsAA, p.(Ala178fs)	30 days	No	Biotin and thiamine	ND
	/c.620delinsAA, p.(Ala178fs)				
Kamasak et al.^18^	c.894T>G, p. (Tyr298*)/-	23 days	No	Biotin and thiamine	Death
Kılıç et al.^19^	p.His200Serfs*,c.(597insThr)	20 days	Yes	Biotin and thiamine	Death
	/p.His200Serfs*, c.(597insThr)				
Kılıç et al.^19^	c.894T>G, p.(Tyr298*)	21 days	Yes	Biotin and thiamine	Quadriplegia
	/c.894T>G, p.(Tyr298*)				

*ND* not described, *NP* not performed.

Although BBGD is a genetic condition that is treatable by initiation of thiamine and biotin treatment, neonatal onset may predict a poor prognosis, as there is only a reported case in which the patient achieved independent gait (Table 1). Furthermore, before biotin and thiamine are initiated, neuronal damage may be too severe to gain favorable intellectual or motor development. As we performed target sequencing, we cannot deny the possibility that variants outside of the targeted genes influenced his development. Nevertheless, vitamin combination therapy may prevent a second attack or encephalopathy, as in our patient.

Treatment before the onset of encephalopathy is required to attain better neurological outcomes. Moreover, elucidation of biomarkers for BBGD may provide a screening method and improve outcomes^13–19^.

## Supplementary information

Supplementary Figure 1

Supplementary Table 1

## Data Availability

The relevant data from this Data Report are hosted at the Human Genome Variation Database at 10.6084/m9.figshare.hgv.3225, 10.6084/m9.figshare.hgv.3228.
