# Supplementary figures and images for: A Japanese patient with neonatal biotin-responsive basal ganglia disease
Source: Hum Genome Var. 2022 Sep 29;9:35. doi: 10.1038/s41439-022-00210-z (PMC9522647; doi:10.1038/s41439-022-00210-z)

## Slide 1
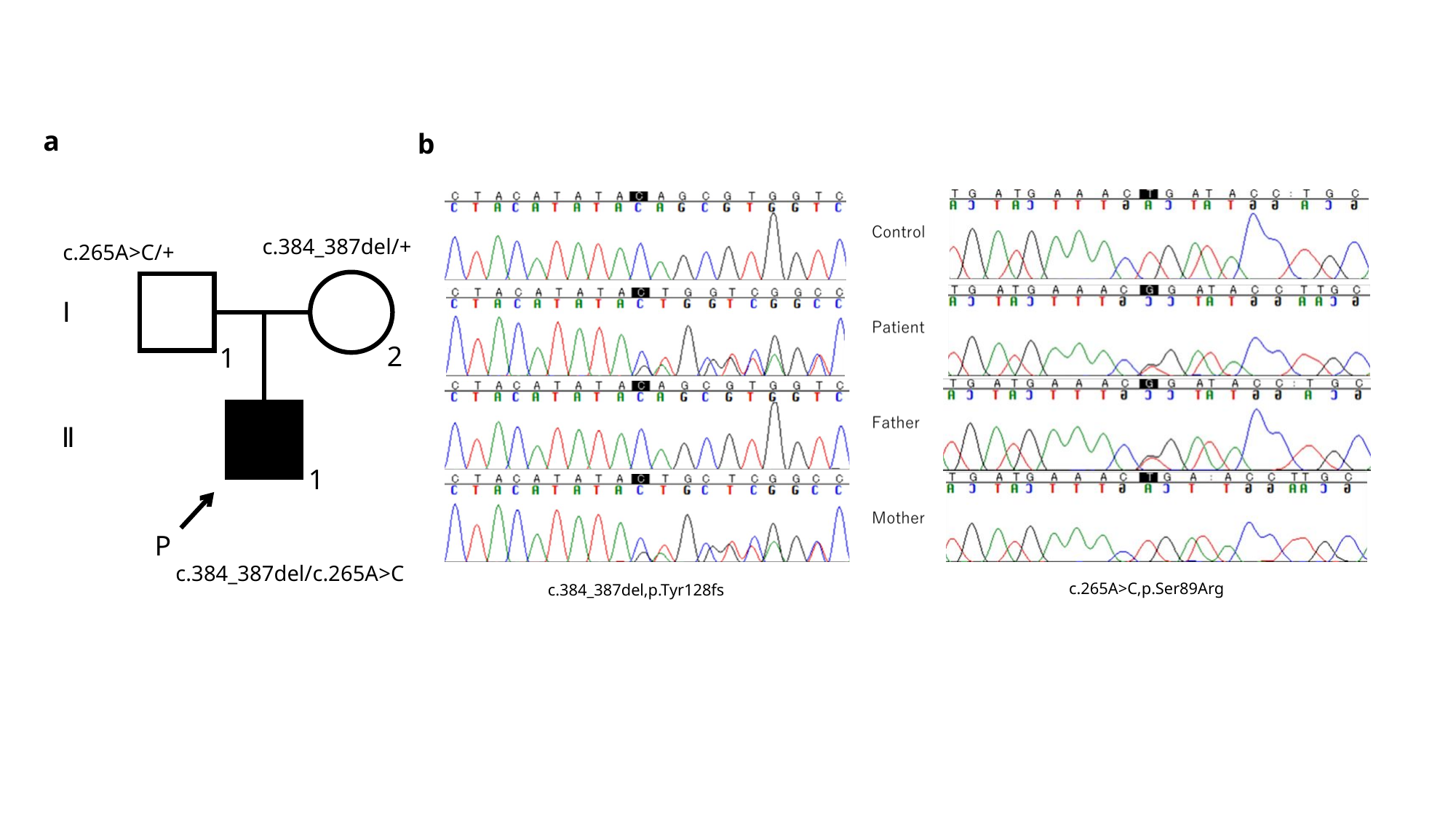

a
b
c.265A>C,p.Ser89Arg
c.384_387del,p.Tyr128fs
c.384_387del/+
c.265A>C/+
Ⅰ
Ⅱ
2
1
1
P
c.384_387del/c.265A>C

Supplement: Supplementary file 1 — Supplementary Figure 1 [file 41439_2022_210_MOESM1_ESM.pptx]
