# Supplementary material for: A Japanese patient with neonatal biotin-responsive basal ganglia disease
Source: Hum Genome Var. 2022 Sep 29;9:35. doi: 10.1038/s41439-022-00210-z (PMC9522647; doi:10.1038/s41439-022-00210-z)
Supplement: Supplementary file 2 — Supplementary Table 1 [file 41439_2022_210_MOESM2_ESM.docx]

**Supplementary Table 1**

ClinVar SI​FT Polyphen２ CADD REVEL Mutation Assessor

c.265A>C,p.(Ser89Arg) Pathogenic​ 0.020 0.877 22 0.62 0.939

deleterious possibly damaging likely benign likely disease causing high
